# Is Body Dysmorphic Disorder Associated with Abnormal Bodily Self-Awareness? A Study Using the Rubber Hand Illusion

**DOI:** 10.1371/journal.pone.0099981

**Published:** 2014-06-12

**Authors:** Ryan A. Kaplan, Peter G. Enticott, Jakob Hohwy, David J. Castle, Susan L. Rossell

**Affiliations:** 1 School of Psychology and Psychiatry, Monash University, Melbourne, Australia; 2 Monash Alfred Psychiatry Research Centre, The Alfred and Central Clinical School, Monash University, Melbourne, Australia; 3 Brain and Psychological Sciences Research Centre, Swinburne University of Technology, Melbourne, Australia; 4 Department of Psychiatry, St. Vincent's Hospital and University of Melbourne, Melbourne, Australia; 5 School of Philosophical, Historical, and International Studies, Monash University, Melbourne, Australia; 6 Cognitive Neuroscience Unit, School of Psychology, Deakin University, Burwood, Australia; University G. d'Annunzio, Italy

## Abstract

Evidence from past research suggests that behaviours and characteristics related to body dissatisfaction may be associated with greater instability of perceptual body image, possibly due to problems in the integration of body-related multisensory information. We investigated whether people with body dysmorphic disorder (BDD), a condition characterised by body image disturbances, demonstrated enhanced susceptibility to the rubber hand illusion (RHI), which arises as a result of multisensory integration processes when a rubber hand and the participant's hidden real hand are stimulated in synchrony. Overall, differences in RHI experience between the BDD group and healthy and schizophrenia control groups (*n* = 17 in each) were not significant. RHI strength, however, was positively associated with body dissatisfaction and related tendencies. For the healthy control group, proprioceptive drift towards the rubber hand was observed following synchronous but not asynchronous stimulation, a typical pattern when inducing the RHI. Similar drifts in proprioceptive awareness occurred for the BDD group irrespective of whether stimulation was synchronous or not. These results are discussed in terms of possible abnormalities in visual processing and multisensory integration among people with BDD.

## Introduction

The rubber hand illusion (RHI), first described by Botvinick and Cohen [1], occurs when a participant's hand is rested on a surface and hidden from view, and a fake hand is placed in view, alongside the real hand. The participant is then directed to watch the fake hand as it, and the hidden real hand, are both touched repeatedly and synchronously. This procedure typically results in a number of illusory experiences for the participant including the experience that the touch felt by the participant is caused by the touch seen on the fake hand, and an illusory feeling of ownership over the fake hand.

While top-down factors, such as the plausibility of the physical characteristics and positioning of the fake hand, may modulate the vividness of the RHI experience [2], the RHI is thought to arise predominantly as a result of the interaction of a number of related sensory processes. These processes include visual capture, multisensory integration, and sensory processing mechanisms specific to the area of space closely surrounding the body, known as peripersonal space [1], [3], [4], [5], [6], [7], [8]. A growing body of research indicates that sensory events within peripersonal space are responded to by specialised neurons capable of multimodal sensory processing, a capability which is thought to facilitate the integration of information from different senses [9], [10], and be crucial to formulating and maintaining a perceptual representation of the body [5].

Compromised body perception is a feature common to a number of psychiatric disorders. These include the eating disorders and body dysmorphic disorder (BDD). BDD is a condition characterised by preoccupation with perceived but non-existent flaws or abnormalities in one's appearance ([11], [DSM-IV-TR],[12]). The nature of the problems in own-body perception in BDD are not well understood, in part because of the inherent challenges in its objective assessment [13]. There is evidence that aberrant processing of visual information may be involved [14], [15], [16], and a possible role for problems in multisensory integration has also been suggested [13]. This contention is supported by the supposed role of multisensory integration in the formation of a perceptual body image [5]. Evidence also suggests that parietal brain regions, which are associated with disturbances in body image and body perception, are also important in multisensory integration [6], [17], [18], [19], [20]. The possible role of parietal brain areas in BDD symptomatology has been previously proposed [13], [21].

Given the roles of visual and multisensory mechanisms in creating the RHI, it is a useful paradigm for furthering our understanding of processes of own-body perception in BDD. Importantly, evidence also implicates the parietal lobe in the RHI [22]. Although, to our knowledge, the RHI has not been previously investigated in BDD, there is some evidence of a relationship between BDD-relevant traits and RHI experience. A study of healthy undergraduate students by Mussap and Salton [23] produced findings suggestive of a positive association between RHI strength, as measured by self-report questionnaire, and behaviours related to unhealthy body development and body image. The authors suggested that this indicated a correspondence between RHI susceptibility and the malleability or instability of one's perceptual body image. Furthermore, Eshkevari, Rieger, Longo, Haggard, and Treasure [24] found self-reported RHI experience to be greater among individuals with eating disorders than healthy controls. They also observed positive relationships between RHI susceptibility and eating disorder-related variables including body dissatisfaction, drive for thinness, and bulimia-related tendencies. Like Mussap and Salton, Eshkevari and colleagues speculated that their findings pointed to greater plasticity in the body representations of individuals with eating disorders, possibly influenced by problems in multisensory integration and non-normative emphasis on visual input in processes of body perception.

Of relevance to investigations of such processes in BDD is that patients can often exhibit schizotypal features, especially delusionality [25], [26], [27], [28]. Previous research findings point to a possible link between RHI experience and positive schizotypal traits in healthy individuals [29], [30]. Individuals with schizophrenia have also been shown to experience the RHI more strongly and quickly than healthy controls, and evidence indicates that RHI strength may be positively associated with hallucinations and delusional experiences, such as delusions of reference, among people with schizophrenia [30], [31]. The mechanisms of this enhanced RHI susceptibility among people high in schizotypy are unclear. However, it has been suggested that it may result from deficits in the integration of multisensory information, and in the process by which multisensory body-related input is reconciled against existing body representations [29], [30].

This study aimed to investigate processes of own-body perception in BDD using the RHI. To do so, we recruited a group of people with BDD and a healthy control group. Since BDD can incorporate schizotypal features, and because of the relationship between schizotypal traits and RHI experience, we also recruited a group of individuals with schizophrenia and schizoaffective disorder as a psychiatric control group. Because of evidence in the literature of greater RHI susceptibility among individuals with body image disorders, and an association between RHI experience and BDD-like behaviours and traits we hypothesised that people with BDD would experience the RHI more strongly than healthy controls. We also hypothesised that there would be a positive correlation between RHI susceptibility and BDD-relevant symptoms and traits, as measured with the Dysmorphic Concern Questionnaire and relevant subscales of the Eating Disorder Inventory 3^rd^ Edition. Finally, we predicted that schizotypal traits, measured using the Perceptual Aberration, Somatic Symptoms, and Social Fear scales, would be positively associated with RHI susceptibility.

## Methods and Materials

### Ethics statement

The study protocol was approved by the Human Research Ethics Committees of Monash University and the Alfred Hospital, Melbourne, and abided by the Declaration of Helsinki. All participants provided written informed consent. Capacity to consent was established by asking participants about the study and assessing whether they could recall and understand what was being asked of them.

### Participants

The sample was made up of three groups each with 17 participants: a BDD group, healthy control group (HC), and schizophrenia/schizoaffective disorder group (SZ). Demographic data for each group are shown in Table 1. Participants for the BDD group were recruited from the St Vincent's Hospital Melbourne Body Image Clinic. Participants for the HC and SZ groups were recruited from a voluntary research participant database and via advertisements placed in university and community newsletters. Posters were also placed in the facilities of public and community mental health services. BDD participants had a current DSM-IV-TR diagnosis of BDD, diagnosed by their treating clinician and confirmed with the Body Dysmorphic Disorder Diagnostic Module (BDD-DM; [32]). SZ participants had a current and primary DSM-IV-TR diagnosis of either schizophrenia or schizoaffective disorder, with no eating disorder history. They were screened using the MINI International Neuropsychiatric Interview version 5.0.0 (MINI; [33]). HC participants had no history of mental illness or neurological injury and were screened using the MINI Screen version 5.0.0 [33].

**Table 1:** RHI Questionnaire items.

Item
1. It seemed as if I were feeling the touch of the experimenter's finger in the location where I saw the rubber hand touched.
2. It seemed as though the touch I felt was caused by the experimenter's finger touching the rubber hand.
3. I felt as if the rubber hand were my hand.
4. It felt as if my (real) [right/left] hand were drifting towards the rubber hand.
5. It seemed as if I might have more than one [right/left] hand or arm.
6. It seemed as if the touch I was feeling came from somewhere between my own hand and the rubber hand.
7. It felt as if my (real) hand were turning ‘rubbery’.
8. It appeared (visually) as if the rubber hand were drifting towards the [right/left] (towards my [right/left] hand).
9. The rubber hand began to resemble my own (real) hand, in terms of shape, skin tone, freckles or some other visual feature.

*Note.* Words in brackets were selected accordingly depending on the side stimulated.

### Measures

#### MINI International Neuropsychiatric Interview version 5.0.0 and MINI Screen version 5.0.0 (MINI; [33])

The MINI is a structured diagnostic interview for psychiatric disorders, with good psychometric properties [33], [34]. The MINI Screen is an abridged version of the MINI, used as a screening tool and to determine whether any symptoms warrant further investigation with the MINI. The MINI and MINI Screen were used to ensure participants met the requirements for inclusion in the relevant group.

#### Body Dysmorphic Disorder Diagnostic Module (BDD-DM; [32])

As the MINI does not assess for BDD, the BDD-DM, a brief structured interview designed to diagnose BDD, was used.

#### Wechsler Test of Adult Reading (WTAR; [35])

The WTAR is a test of premorbid IQ in which participants read aloud a list of 50 words with unusual spellings and are scored based on correct pronunciation of the words. The WTAR was co-normed with the Wechsler Adult Intelligence Scale (WAIS) and WTAR scores are therefore used to predict full-scale WAIS IQ. It was used to ensure estimated mean IQ scores were equivalent across the groups.

#### Edinburgh Handedness Inventory (EHI; [36])

The EHI, a 22-item self-report measure, was used to match groups on handedness.

#### Depression, Anxiety, and Stress Scales, 42-item version (DASS-42; [37])

To control for depression and anxiety, both of which commonly occur comorbidly with BDD and SZ, the DASS was used. The DASS is a 42-item self-report measure that produces a score for each of the three clinical symptom dimensions of depression, anxiety, and stress. Respondents endorse items to do with depression-, anxiety-, and stress-related symptoms on a four-point Likert scale according to how frequently they experienced those symptoms over the prior week. The DASS-42 has sound psychometric properties [38]. One participant failed to complete the second page and, as per the authors' guidelines, her responses from page 1 were adjusted appropriately and used in lieu.

#### Yale-Brown Obsessive Compulsive Scale Modified for BDD (BDD-YBOCS; [39])

The BDD-YBOCS assesses the extent of cognitive and behavioural preoccupation with appearance defects. It was used to assess BDD symptom severity in the BDD group. It is used widely in research and clinical work for this purpose. The BDD-YBOCS is a 12-item interview-style questionnaire, administered by the researcher or clinician, employing a five-point Likert scale. The scores for all items are summed to create a total score of BDD severity.

#### Dysmorphic Concern Questionnaire (DCQ; [40])

The DCQ is a seven-item self-report questionnaire using a Likert-type scale. The DCQ assesses severity of BDD-related concerns and behaviours across a spectrum including nil concerns, normative non-pathological appearance-related levels of concern, and clinically significant body dysmorphic concerns.

#### Scale for the Assessment of Positive Symptoms (SAPS; [41]) and Scale for the Assessment of Negative Symptoms (SANS; [42])

To control for variability in schizophrenia symptom severity within the SZ group, the SAPS and SANS, both structured clinical interviews, were used to assess positive and negative psychosis symptoms respectively.

#### Perceptual Aberration Scale [43], Somatic Symptoms Scale [44], and Social Fear Scale [45]

These self-report scales were used to assess for schizotypal characteristics relating to perceptual and bodily experiences, and asocial drives.

#### Eating Disorder Inventory, 3^rd^ Edition (EDI-3; [46])

The EDI-3 is a self-report measure consisting of 91 items, each a statement that requires endorsement on a six-point Likert scale. The EDI-3 produces scale scores for a number of eating disorder-relevant characteristics. Of relevance to this study were the subscales pertaining to tendencies and behaviours associated with body dissatisfaction. These are Body Dissatisfaction (BD), Bulimia (B), and Drive for Thinness (DT).

#### RHI Questionnaire

To assess subjective illusion experience, we used the nine-item questionnaire used in Botvinick and Cohen's [1] original RHI study. This questionnaire and variants of it have been used extensively in RHI research. Each questionnaire item consists of a statement reflecting an anomalous perceptual experience that may plausibly arise as a result of the RHI procedure. Items are shown in Table 1. Participants endorse each statement on a seven-point Likert scale ranging from -3 (“strongly disagree”) to +3 (“strongly agree”). Different scoring methods have been used in the literature, with some authors analysing the results of each item individually and others creating an index score based on the responses to all nine items. A commonly used method is to create an index score using only the first three items, which tend to be most consistently and strongly endorsed and are considered to most accurately reflect illusion experience (e.g. [47], [48], [49], [50], [51]) The remaining six items, which tend to be endorsed only minimally among healthy samples, are used to control for suggestibility and task demand characteristics (e.g. [48], [49], [51]).

#### Proprioceptive Drift

Based on early findings that appeared to show that the RHI resulted in a shift of proprioceptive awareness towards the rubber hand, participant post-trial judgments of the felt location of their stimulated hidden hand relative to either a baseline judgment or actual hand position, known as “proprioceptive drift”, has been widely used as a measure of illusion strength [6]. Although there is now more evidence to support the notion that changes in felt hand position result from the RHI procedure, research has also shown that this effect may be uncorrelated with illusion experience and may operate via different mechanisms (for example [52], [53]). We included a measure of proprioceptive drift in this study as a means of evaluating the effect of the RHI on proprioceptive awareness in our sample, and to allow for comparison with other studies that have employed similar measures.

To measure participants' felt hand location, a cover was placed over the experimental apparatus and the experimenter slid a pointer along the top edge of the cover, beginning from the outer edge of the box in which the participant's stimulated hand rested. The speed at which the pointer was moved was varied so as to avoid predictability and prevent any between-trial carryover effects. The participant was asked to tell the experimenter to stop when the pointer was in line with where the index finger of the stimulated hand was felt to be. The experimenter then read the pointer position off a ruler attached to the cover but out of view of the participant. This was done at baseline and after each trial. Proprioceptive drift was calculated as the participant's post-trial judgment minus their judgment at baseline.

### Procedure

**Figure 1:**
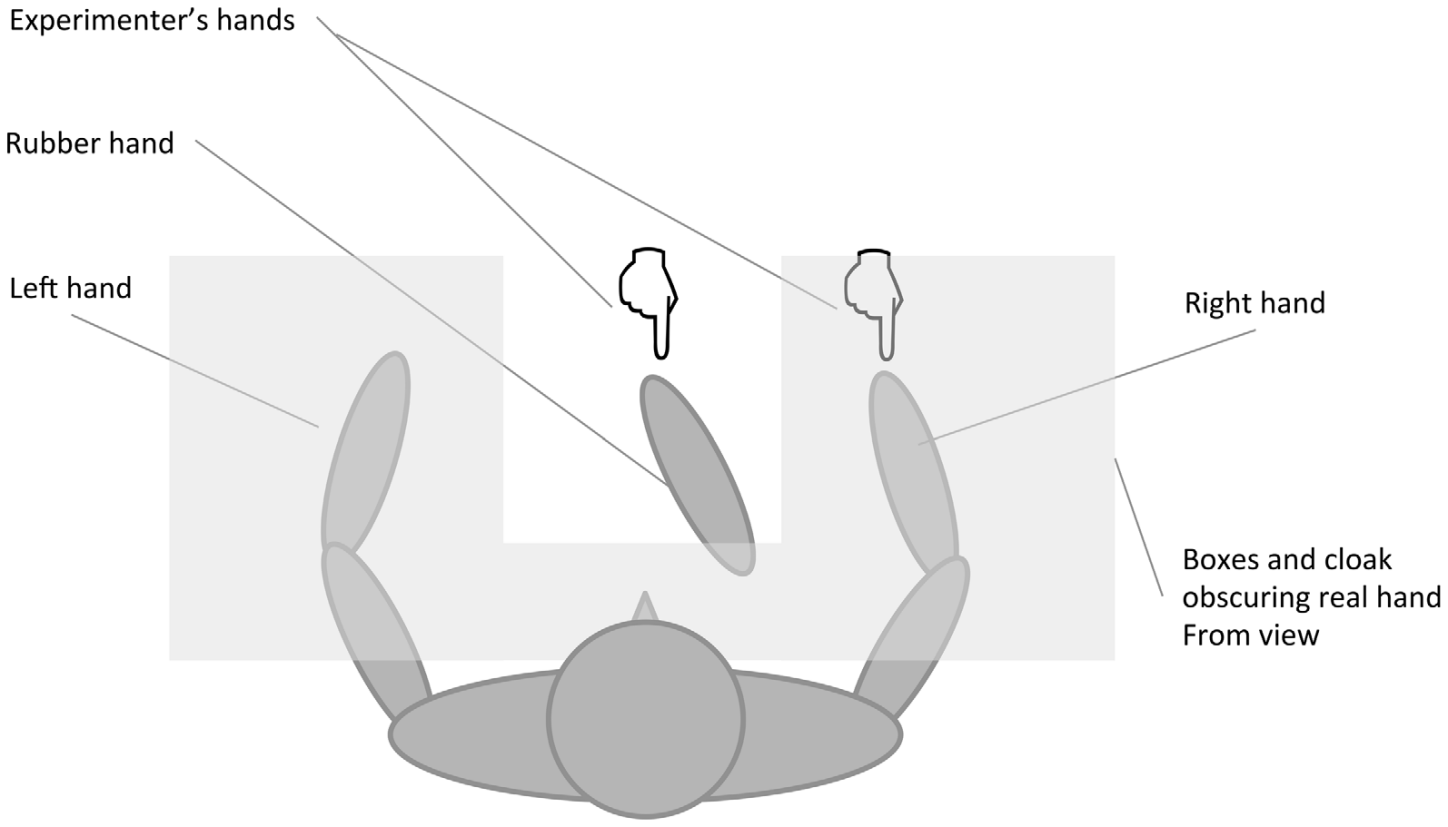
Conceptual diagram of the RHI apparatus setup.

After providing signed informed consent, participants completed the self-report measures. The other measures were then administered. The RHI procedure followed. For this procedure, the participant was seated in front of the RHI apparatus, which consisted of two wooden boxes (width 50 cm; depth 60 cm; height 20 cm), set apart by 40 centimetres (see Figure 1). The participant's chair was positioned such that their midline aligned with the midpoint between the two boxes. The participant was then instructed to place their hands and forearms inside the boxes, palm down. A black cloak was used to cover the participant's front and upper arms. The baseline proprioceptive judgment for each hand was obtained, as described above, and the apparatus cover was then removed. Each participant underwent four stimulation trials: a synchronous and asynchronous trial for each hand. Hand order was counterbalanced across participants, as was stimulation mode. Before each trial, a lifelike prosthetic hand corresponding to the hand being tested was placed on the surface between the two boxes and positioned so as to be posturally congruent with the participant's real hand. Masculine- or feminine-looking prostheses were used for male and female participants respectively. The experimenter then began the stimulation. The experimenter stimulated the real and rubber hands by stroking them with his fingers in corresponding locations. During the synchronous trials, strokes were administered at intervals of approximately 500 milliseconds. In asynchronous trials, there was a delay of approximately 500 milliseconds between strokes administered to the real and fake hands. In each trial, stimulation continued for a total of 4.5 minutes, with breaks of 15 seconds duration after 90 seconds and 180 seconds during which temperature recordings of the participant's hands were taken (temperature data are not reported here). The cover was then replaced and a post-trial proprioceptive judgment was obtained. The participant was then asked to complete the RHI questionnaire. The procedure was then repeated on the same hand for the other stimulation mode, and then again for the two stimulation modes with the other hand.

### Data Analysis

RHI questionnaire scores were transformed by adding three to each score so that the possible range of scores was 0 to 6 instead of −3 to +3. Data for each item of the RHI questionnaire were then subjected to a 3 (group) ×2 (side) ×2 (stimulation mode) mixed model ANOVA. We then created an illusion index score and control index score for each participant. This was done by computing the mean of items 1–3 for the illusion score, and the mean of items 4–9 for the control score. These scores were then analysed using a 3 (group) ×2 (side) ×2 (stimulation mode) ×2 (index) mixed model ANOVA. Proprioceptive drift scores were entered into a 3 (group) ×2 (side) ×2 (stimulation mode) mixed model ANOVA. Because of the factorial design of the study and the number of factors, ANOVAs were used despite some cases of non-normality. All results were confirmed with non-parametric tests. Non-parametric tests (either Wilcoxon or Mann-Whitney tests as appropriate) were used for all post-hoc analyses. Spearman correlational analyses were undertaken to investigate relationships between RHI experience, proprioceptive drift, and clinical/symptom variables. Alpha was set at .05 for primary analyses. A more conservative alpha of .01 was used in the case of post-hoc tests.

## Results

### Demographic and clinical data

Shown in Table 2 are means and standard deviations for the clinical and demographic variables for each group. The three groups were equivalent in terms of average age, handedness score, premorbid IQ, and sex distribution. As expected, the BDD and SZ groups had significantly higher mean depression, anxiety, and stress scores than the HC scores. Scores on BDD symptom measures (DCQ and BDD-YBOCS) were significantly higher in the BDD group than the other two groups. Scores on the measures of schizophrenia symptomatology (SAPS and SANS) were significantly higher in the SZ than the other two groups. There were no significant group differences on the three EDI-3 scales.

**Table 2:** Demographic and clinical data.

Variable	BDD	SZ	HC	Group comparison	
Sex, *n*					
Males	4	4	4		
Females	13	13	13		
Age Range, *years*	18–62	24–59	23–61		
Age	36.41 (11.27)	39.53 (9.97)	35.41 (9.73)	*F*(2,48) = 0.73, *p* = .49	
EHI	70.84 (36.86)	71.70 (43.22)	72.93 (28.67)	*H* = 0.20, *p* = .90	
WTAR	97.47 (9.86)	100.00 (12.86)	101.88 (16.00)	*H* = 2.85, *p* = .24	
DASS Depression	16.18 (11.56)	12.94 (12.15)	1.59 (1.70)	*H* = 16.98, *p*<.001	(BDD = SZ) >HC
DASS Anxiety	9.12 (7.93)	10.12 (10.12)	1.53 (1.63)	*H* = 17.08, *p*<.001	(BDD = SZ) >HC
DASS Stress	17.59 (12.58)	15.06 (11.29)	5.71 (3.65)	*H* = 10.58, *p* = .005	(BDD = SZ) >HC
DCQ	17.00 (3.64)	7.47 (5.67)	2.94 (1.95)	*H* = 31.14, *p*<.001	(BDD>SZ) >HC
BDD-YBOCS	24.53 (10.25)	2.88 (7.94)	-	*U* = 14.00, *p*<.001	BDD>SZ
SAPS	1.76 (2.93)	12.41 (13.87)	-	*U* = 61.00, *p* = .003	BDD<SZ
SANS	0.82 (2.68)	9.94 (10.56)	-	*U* = 46.50, *p*<.001	BDD<SZ
Social Fear	17.00 (8.02)	14.47 (10.38)	6.47 (7.57)	*H* = 13.29, *p* = .001	(BDD = SZ) >HC
Somatic Symptoms	9.29 (7.19)	11.76 (8.03)	5.12 (4.72)	*H* = 6.72, *p* = .035	(BDD = SZ) >HC
Perceptual Aberration	5.06 (3.03)	6.29 (5.18)	2.24 (2.11)	*H* = 10.58, *p* = .005	BDD = (SZ<HC)
**EDI3 Scales**					
Drive for Thinness	11.12 (9.23)	10.00 (6.73)	6.12 (5.71)	*H* = 3.15, *p* = .21	
Bulimia	6.29 (8.85)	6.65 (6.15)	3.82 (5.34)	*H* = 1.46, *p* = .48	
Body Dissatisfaction	17.76 (12.22)	18.65 (10.75)	12.88 (9.35)	*H* = 2.53, *p* = .28	

*Note.* All figures are group means, with standard deviations in parentheses, unless otherwise stated. BDD =  body dysmorphic disorder group; SZ =  schizophrenia/schizoaffective disorder group; HC =  healthy control group; EHI =  Edinburgh Handedness Inventory; WTAR =  Wechsler Test of Adult Reading; DASS =  Depression, Anxiety, and Stress Scales; DCQ =  Dysmorphic Concern Questionnaire; BDD-YBOCS = BDD-modified Yale-Brown Obsessive Compulsive Scale; SAPS =  Scale for the Assessment of Positive Symptoms; SANS =  Scale for the Assessment of Negative Symptoms; EDI3 =  Eating Disorder Inventory, 3^rd^ Edition. Except for age, comparisons were performed using either Kruskal-Wallis or Mann-Whitney tests due to violations of normality and homogeneity of variance.

### RHI Questionnaire

#### Individual items

**Figure 2:**
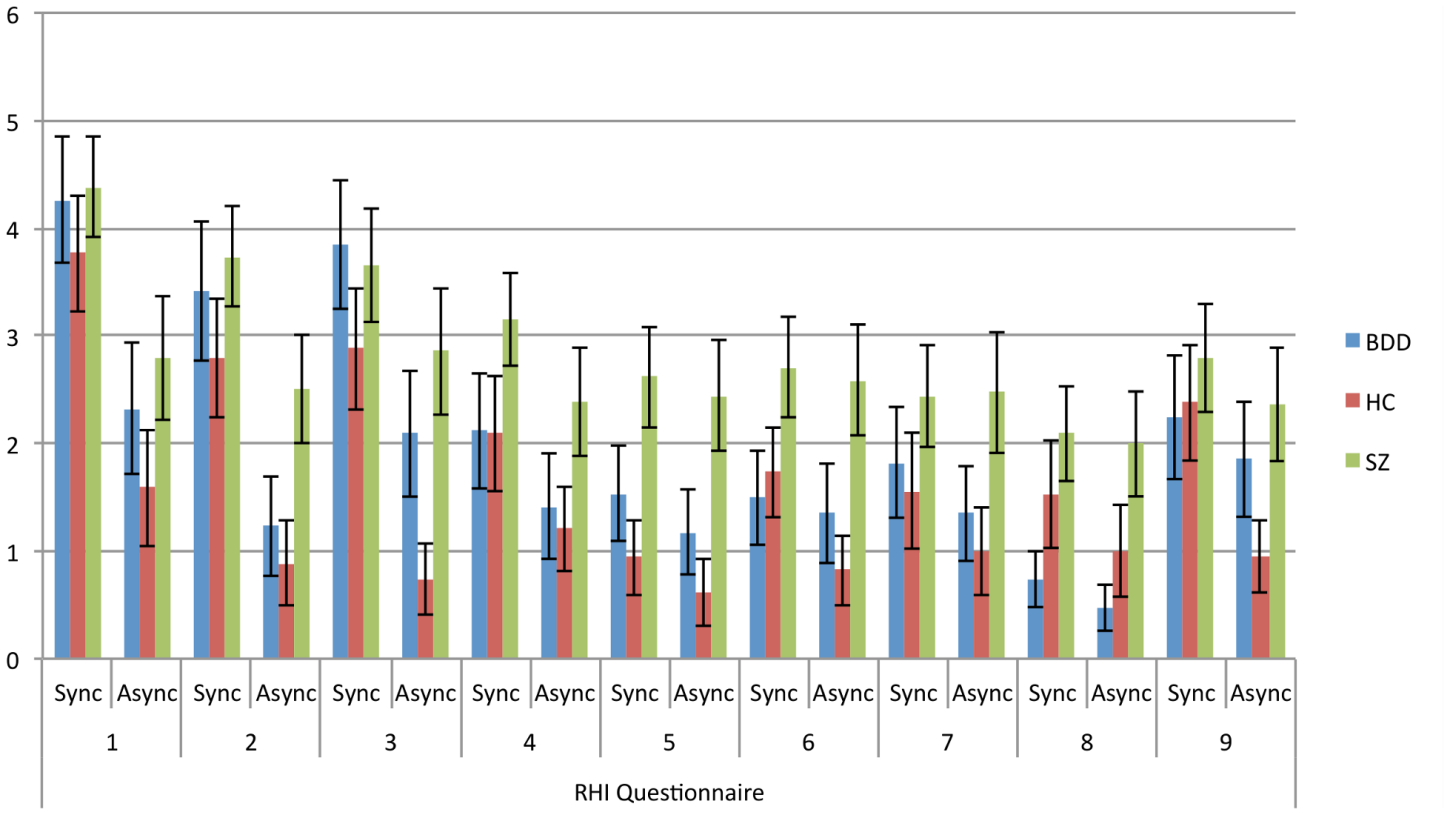
Means of scores for left and right sides for each RHI questionnaire item. Error bars indicate ±1 standard error.

Means and standard deviations for each item for each group are shown in Table 3. Illustrated in Figure 2 are group means for scores on the left and right sides in the different stimulation conditions. The three-way ANOVAs showed a main effect of stimulation mode for all nine items, with scores in the synchronous stroking conditions greater than scores in the asynchronous conditions for each item. There were main effects of group for items 5, 6, and 8. These reflected that the SZ group had the highest scores on these items. However, only the difference between the SZ and HC groups for item 5 (*p* = .002), and between the SZ and BDD groups for item 8 (*p* = .004) achieved statistical significance at alpha  = .01. The difference between the SZ and HC groups for item 2 fell short of significance (*p* = .016). For item 9, there was a main effect of side, with scores for the right hand greater than scores for the left hand [*F*(1,48) = 4.11, *p* = .048, *η*_p_^2^ = .08]. In addition, there was a group-by-stimulation mode interaction for item 9 [*F*(2,48) = 3.56, *p* = .036, *η*_p_^2^ = .13]. For the HC group, scores on item 9 were greater in the synchronous than the asynchronous conditions [*Z* = 2.94, *p* = .003], but this was not the case for the BDD [*Z* = 1.13, *p* = .26] and SZ groups [*Z* = 1.31, *p* = .19].

**Table 3:** Means (standard deviations) for each index/condition combination by group.

Index/Condition	BDD	SZ	HC	Group comparison
Illusion Index				
Synchronous	3.84 (2.04)	3.92 (1.51)	3.15 (1.99)	BDD = SZ = HC
Asynchronous	1.88 (1.48)	2.72 (1.76)	1.07 (1.38)	BDD = (SZ>HC)
Control Index				
Synchronous	1.66 (1.49)	2.63 (1.46)	1.71 (1.62)	BDD = SZ = HC
Asynchronous	1.27 (1.37)	2.37 (1.80)	0.93 (1.25)	BDD = (SZ>HC)

*Note.* All figures are group means of left hand and right hand scores, with standard deviations in parentheses.

#### Index scores

The four-way ANOVA examining illusion and control index scores showed that scores for the items reflecting illusion experience (the illusion index) were significantly greater than for items in the control index [*F*(1,48) = 49.22, *p*<.001, *η*_p_^2^ = .51]. This indicates that across the whole sample, the RHI procedure yielded the expected response pattern. There was also a main effect of stimulation mode, with synchronous stroking yielding higher scores than asynchronous stroking [*F*(1,48) = 53.21, *p*<.001, *η*_p_^2^ = .53]. The main effect of group was also significant [*F*(2,48) = 3.21, *p* = .049, *η*_p_^2^ = .12]. Post hoc tests showed scores were greater for the SZ group than the HC group (*p* = .04), but this difference did not achieve statistical significance at alpha  = .01. There was no main effect of side. A significant stimulation mode-by-index interaction [*F*(1,48) = 47.70, *p*<.001, *η*_p_^2^ = .50] indicated that scores were greatest for the illusion index for synchronous stroking trials, and lowest for the control index for asynchronous stroking trials. There were no other significant interactions.

#### Correlations

To examine associations between illusion strength and clinical/demographic variables, Spearman correlation coefficients were computed. We used the illusion index scores for the synchronous trials as the illusion strength variable in these analyses. To minimise the likelihood of type I error, an alpha of .01 was used. Correlation coefficients are shown in Table 4. Across the whole sample, there were positive correlations between illusion strength and variables relating to BDD symptoms and traits. There were also positive correlations between illusion strength scores and scores on the Social Fear Scale.

**Table 4:** Spearman correlation coefficients for self-reported illusion strength, proprioceptive drift, and clinical variables.

	Prop. Drift	DASS-D	DASS-A	DASS-S	SOM	SFS	PAS	DCQ	EDI-DT	EDI-B	EDI-BD
Illusion strength (questionnaire)	.325**	.190	.213	.200	.165	.330*	.070	.253^#^	.303^#^	.419*	.388*
Proprioceptive drift		−.180	.004	−.028	−.089	.228^†^	.200^†^	.192^†^	−.032	.107	.106

* *p*<.01, one-tailed; ^#^ *p*<.05, one-tailed; ^†^ *p*<.10, one-tailed

*Note.* DASS-D = DASS Depression Scale; DASS-A = DASS Anxiety Scale; DASS-S = DASS Stress Scale; SOM =  Somatic Symptoms Scale; SFS =  Social Fear Scale; PAS =  Perceptual Aberration Scale; DCQ =  Dysmorphic Concern Questionnaire; EDI-DT =  EDI3 Drive for Thinness Scale; EDI-B =  EDI3 Bulimia Scale; EDI-BD =  EDI3 Body Dissatisfaction Scale.

With regards to the group-specific variables, for the BDD group there was not a significant association between illusion strength and scores on the BDD-YBOCS [*ρ* = −.01, *p* = .49, one-tailed]. For the SZ group, correlations between illusion strength and scores on the SANS [*ρ* = .31, *p* = .22, two-tailed] and SAPS [*ρ* = .14, *p* = .60, two-tailed] were both non-significant.

### Proprioceptive Drift

**Figure 3:**
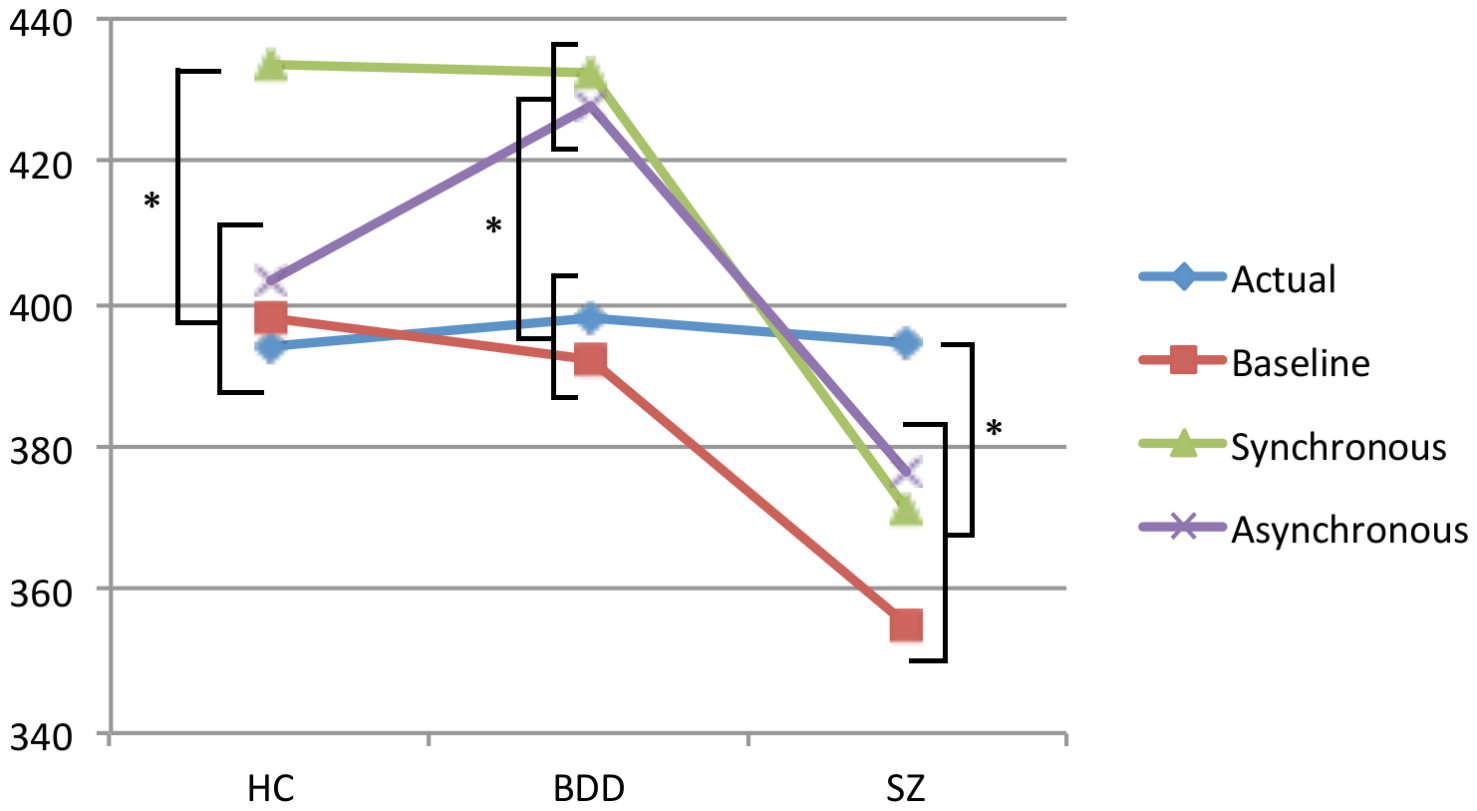
Mean actual hand position and proprioceptive judgment for each group at baseline and following synchronous and asynchronous stimulation trials. * *p*<.05

The three-way ANOVA revealed that there was no significant main effect either of group or of side stimulated. Proprioceptive drift scores were greater in synchronous (*M* = 30.50, *SE* = 9.48) compared to asynchronous (*M* = 20.61, *SE* = 7.53) stroking conditions, but the main effect of stimulation mode fell short of statistical significance [*F*(1,48) = 3.54, *p* = .066, *η*_p_^2^ = .07]. The group-by-stimulation mode interaction was significant [*F*(2,48) = 4.09, *p* = .023, *η*_p_^2^ = .15]. This interaction can be seen graphically in Figure 3, along with plots of actual hand position and baseline proprioceptive position estimates (means for both sides).

As Figure 3 shows, for the HC group, proprioceptive drift following synchronous stroking was greater than following asynchronous stroking (“proprioceptive shift”). However, the difference did not reach statistical significance at our adjusted alpha [*Z* = 2.07, *p* = .038, two-tailed]. Whereas the proprioceptive judgments at baseline and after asynchronous stroking were similar [*Z* = 0.71, *p* = .48, two-tailed], there was a significant difference between the judgments at baseline and after synchronous stroking [*Z* = 2.68, *p* = .007, two-tailed]. For the BDD group, proprioceptive shift was negligible, with similar proprioceptive drift in synchronous and asynchronous conditions [*Z* = 1.22, *p* = .23, two-tailed]. Additionally, in the BDD cohort, proprioceptive estimates were significantly different from baseline following both synchronous [*Z* = 2.72, *p* = .006, two-tailed] and asynchronous [*Z* = 2.63, *p* = .009, two-tailed] stroking. For the SZ group, although proprioceptive estimates were similar irrespective of stimulation mode [*Z* = −1.19, *p* = .23, two-tailed], neither was significantly different from baseline, with *Z* = 1.02, *p* = .31, two-tailed, for the synchronous difference, and *Z* = 1.14, *p* = .26, two-tailed, for the asynchronous difference.

To understand whether there were group differences in proprioceptive judgment in the absence of any hand stimulation, the difference between participants' baseline estimates of hand position and actual hand position for each hand were computed. The means of these difference scores for the left and right hands were then entered into a one-way independent measures ANOVA which revealed an effect of group, [*F*(2,48) = 3.76, *p* = .03, *η*_p_^2^ = .14]. This reflected a significant difference between the HC and SZ groups, *U* = 68.00, *p* = .008, two-tailed. The difference between BDD and SZ groups was not significant, *U* = 87.00, *p* = .048, two-tailed.

#### Correlations

As with the questionnaire data, in the correlational analyses of the proprioceptive drift data we used the proprioceptive drift scores for the synchronous trials only, and an alpha of .01. Spearman correlation coefficients are shown in Table 4. Across the whole sample, there were no significant correlations between proprioceptive drift and the clinical variables. There were, however, weak relationships which fell short of statistical significance between proprioceptive drift and scores on the Social Fear Scale, Perceptual Aberration Scale, and the Dysmorphic Concern Questionnaire. The relationship between proprioceptive drift and RHI questionnaire illusion index scores was non-significant at our adjusted alpha [*ρ* = .33, *p* = .02, two-tailed].

Additionally, for the BDD group, proprioceptive drift was not significantly correlated with scores on the BDD-YBOCS [*ρ* = −.09, *p* = .49, one-tailed]. There were also no significant correlations among SZ participants between proprioceptive drift and scores on the SANS [*ρ* = -.08, *p* = .78, two-tailed] or SAPS [*ρ* = −.23, *p* = .37, two-tailed].

## Discussion

The aim of this study was to gain further insight into own-body perception in BDD using the RHI. The results do not support our prediction of a stronger illusion experience, on average, among the BDD group than the HC group. We did, however, observe moderate and significant positive correlations between self-reported RHI strength and variables relating to BDD-relevant symptoms and traits. These variables were scores on the bulimia, and body dissatisfaction scales of the EDI-3. Positive correlations between illusion strength and scores on the Dysmorphic Concern Questionnaire and EDI-3 drive for thinness scale were significant at alpha  = .05 but non-significant at our adjusted alpha of .01.

The observed associations between RHI strength and tendencies related to appearance concerns are consistent with the findings reported by Eshkevari et al. [24] who found similar correlations in a sample comprising healthy controls and people with eating disorders. Our findings are also partially consistent with those of Mussap and Salton [23] who reported an association between self-reported illusion strength for the left hand and scores on the EDI-3 bulimia scale (although not the body dissatisfaction or drive for thinness scales) in a sample of healthy university students. They also reported a correlation between left hand illusion strength and unhealthy body development behaviours among their male participants only. Our findings therefore support the notion put forward by both Mussap and Salton, and Eshkevari et al., that higher levels of body-related concerns may reflect or be related to a more malleable or plastic perceptual body representation. However, any such relationship may be independent of pathological body concerns, and may rather represent a vulnerability to body-related psychopathology. This is suggested by the fact that our BDD and HC groups did not differ significantly in terms of illusion experience, and that there was no correlation within the BDD group between illusion strength and scores on the BDD-YBOCS.

Our hypothesis of a relationship between illusion strength and schizotypal traits was supported only for scores on the social fear scale. Social evaluation concerns and social withdrawal have been associated with negative body image [54], [55]. Social anxiety and avoidance is also common among people with BDD [56]. People high in social fear and anxiety may therefore have a perceptual body image that is less stable and more susceptible to external influences, which may explain the observed correlation. It is possible, however, that the observed correlation reflects greater suggestibility or influence by task demand characteristics with increasing social anxiety. Interestingly, scores on the other two measures of schizotypal traits were uncorrelated with illusion strength, which is inconsistent with the results of previous studies [29], [30]. Those studies, however, employed broader measures of schizotypy, whereas we selected measures with the specific purpose of assessing body- and perception-related schizotypal traits. This suggests that while RHI experience may indeed be linked to schizotypy, schizotypal cognitive and information processing factors may carry more weight in modulating the experience of the RHI than somatic and perceptual factors.

Of particular interest are our findings relating to item 9 of the RHI questionnaire. Item 9 refers to the experience of the rubber hand beginning to visually resemble the participant's real hand. Although, in the literature, this particular illusory experience does not generally occur with the same reliability and intensity as the sense of ownership over the rubber hand and the sense of referred touch, it often elicits affirmative responses following synchronous stroking of the real and rubber hands (e.g. [1], [57], [58]). Our findings show that participants in the HC group endorsed item 9 more strongly following synchronous than asynchronous stroking, suggesting either that synchronous stroking facilitated that experience, or asynchronous stroking attenuated it. However, there was no such effect of stimulation mode for participants in the BDD and SZ groups. Although speculative, it is possible that for the BDD group, this result may be attributable to the abnormal processing of visual input, especially body-related stimuli. Evidence of idiosyncratic visual processing among people with BDD, suggestive of a focus on details rather than holistic features, has been reported previously [14], [15], [16]. It may be that much in the same way that efficient face recognition relies on processing of configural cues [59], in viewing the rubber hand, BDD participants employ a detail-oriented approach without the holistic integration of the features, and the rubber hand consequently appears less distinct from their own, irrespective of stimulation mode. Alternatively, a history of frequent and prolonged mirror-gazing in people with BDD [60] may affect neural visual processing pathways involved in the processing of body-related visual stimuli. Importantly, neither of these explanations account for the response pattern to this item among the SZ participants. However, it may reflect a tendency of SZ participants to endorse items similarly for synchronous and asynchronous conditions across most of the control items (see Figure 2).

With regards to the proprioceptive drift results, the non-significant relationship between illusion strength and proprioceptive drift scores is in accordance with some previous studies [50], [52] but inconsistent with other findings [1], [24], [61], [62]. Moreover, the absence of a relationship between proprioceptive drift and schizotypal traits is in accordance with findings reported by Germine et al. [29]. Recent research has cast doubt on the validity of proprioceptive drift as a proxy measure of illusion experience [52], [53] and as a result, we have not described it as such. Changes in proprioceptive awareness do seem to result from the RHI procedure, however [52], [53], and the particular pattern of findings across the three groups in the present study is intriguing. The results of the HC group indicate proprioceptive drift towards the rubber hand following synchronous stroking conditions but not asynchronous stroking conditions. Similar patterns have been reported previously [49], [50], [51], [63] (Ehrsson, et al, 2008; Slater, Perez-Marcos, Ehrsson, & Sanchez-Vives, 2008; but see Morgan, et al., 2011; Paton, Hohwy, & Enticott, 2012). In the BDD group, proprioceptive drift occurred to a similar extent as a result of both synchronous and asynchronous stroking, and drift following both types of stimulation was similar to that observed among the HC group following synchronous stroking. Rohde, Di Luca, and Ernst's findings indicate that among healthy participants, proprioceptive drift tends to occur similarly as a result of synchronous stimulation of the fake and real hands, and in the absence of any stroking (i.e. following trials in which participants simply observe the fake hand without it or the real hand being stimulated). Rohde et al. suggested that, in healthy samples, rather than synchronous stroking facilitating proprioceptive drift, asynchrony between the seen and felt stroking disrupts the multisensory integration process that would otherwise occur—a multisensory integration process that weights visual input more favourably than proprioceptive input in cases of conflict (visual capture), thereby resulting in proprioceptive drift towards the seen hand position. Our results may be taken to indicate that, in relation to proprioceptive and bodily awareness, people with BDD are less affected by the stimulation asynchrony than healthy controls. A possible explanation is a difference in multisensory processing involving vision, such that compared to healthy individuals, people with BDD either place greater emphasis on visual information or are more sensitive to it. Indeed, as already noted, evidence points to idiosyncratic processing of visual detail among people with BDD [14], [15], [16].

The results for the SZ group are also interesting. Firstly, the mean baseline estimate for the SZ group was significantly different to that of the HC group, and further away from the midline. This may reflect previously reported deficits in proprioceptive awareness in psychotic illnesses [64], [65], [66], [67], [68], [69]. Secondly, like the BDD group, mean drift towards the rubber hand from the position estimated at baseline was similar in both synchronous and asynchronous conditions. The RHI procedure therefore appears to have had an effect on proprioceptive awareness among the SZ participants, but this was not dependent on synchrony of the stimulation of the real and rubber hands. As with the BDD group, this may indicate differences in multisensory integration in people with psychotic illnesses.

Two additional considerations must be made in interpreting these proprioceptive drift findings. First, the effect of the RHI procedure on proprioceptive awareness and localisation may be influenced by the participant's sense of self. The influence of one's sense of self, or “ipseity”, on RHI experience was proposed by Ferri et al. [70], who found that schizophrenia participants reported significantly weaker feelings of ownership over the rubber hand than healthy controls, in an RHI version in which participants saw the experimenter's hand approaching but not actually stimulating the rubber hand. The authors noted that disturbances of ipseity have been theorised to be a core characteristic of schizophrenia (see [71]) and as such, may help explain why people with schizophrenia may experience and be affected by the RHI differently to healthy individuals.

Second, emerging evidence points to the modulating effect of interoception, or perception of one's internal bodily state, on subjective RHI experience and proprioceptive drift [72], [73]. For example, Tsakiris, Tajadura-Jimenez, and Costantini [72] found that healthy participants' interoceptive sensitivity corresponded to RHI experience, and particularly, proprioceptive drift. On average, participants who demonstrated high interoceptive sensitivity had lower proprioceptive drift estimates than those with low interoceptive sensitivity. Additionally, Eshkevari et al. [24] found proprioceptive drift to be positively correlated with scores on a self-report measure of interoception problems, and that such problems were more prominent in individuals with eating disorders than healthy controls. Future investigation of interoception in BDD, and the relationship between interoception and both RHI experience and proprioceptive drift specifically in schizophrenia and BDD may therefore be useful in further understanding the pattern of results. Furthermore, our SZ proprioceptive drift findings differ from those reported by Thakkar et al. [30], and replication is therefore required to draw inferences with confidence.

Limitations of this study include the relatively small sample size, and comorbidities in both the SZ and BDD groups. However, comorbid or secondary diagnoses are typically seen in such mental illnesses [74], [75] and our samples are therefore representative of the populations from which they have been drawn. Additionally, although reliance on a subjective self-report measure of the RHI is not ideal, objective evaluation of a perceptual experience like the RHI is difficult, if not impossible, and so we have employed a widely-used self-report questionnaire along with sound experimental controls. We also note that in obtaining the proprioceptive drift estimates, the experimenter was not blind to the stimulation condition.

Our findings imply that overall, people with BDD do not differ from healthy individuals in their experience of the rubber hand illusion. However, characteristics and behaviours associated with body dissatisfaction may reflect perceptual body image instability, and may represent a vulnerability to body image-related psychopathology. Moreover, aberrant visual processing in BDD may contribute to inaccuracies in the perception of the positioning and arrangement of body parts. Future research should seek to enhance understanding of the interaction between vision and other sensory input in multisensory integration paradigms, clarify the role of visual processing abnormalities in the development and maintenance of BDD, and explore any implications for treatment.
